# Evaluation of commonly used analysis strategies for epigenome- and transcriptome-wide association studies through replication of large-scale population studies

**DOI:** 10.1186/s13059-019-1878-x

**Published:** 2019-11-14

**Authors:** Jeroen van Rooij, Pooja R. Mandaviya, Annique Claringbould, Janine F. Felix, Jenny van Dongen, Rick Jansen, Lude Franke, BIOS consortium, Peter A. C. ’t Hoen, Bas Heijmans, Joyce B. J. van Meurs

**Affiliations:** 1 Department of Internal Medicine, Erasmus Medical Center, Rotterdam, the Netherlands; 2 Maastricht Centre for Systems Biology (MaCSBio), Maastricht University, Maastricht, the Netherlands; 3 Faculty of Medical Sciences, University of Groningen, Groningen, the Netherlands; 4 The Generation R Study Group, Department of Epidemiology, Erasmus Medical Center, Rotterdam, the Netherlands; 5 The Generation R Study Group, Department of Pediatrics, Erasmus Medical Center, Rotterdam, the Netherlands; 6 Department of Biological Psychology, Vrije Universiteit Amsterdam, Amsterdam, the Netherlands; 7 Department of Psychiatry, VU University Medical Center, Amsterdam, the Netherlands; 8 Department of Genetics, University of Groningen, Groningen, the Netherlands; 9 Department of Human Genetics, Leiden University Medical Center, Leiden, the Netherlands; 10 Centre for Molecular and Biomolecular Informatics, Radboud Institute for Molecular Life Sciences, Radboud University Medical Center Nijmegen, Nijmegen, the Netherlands; 11 Molecular Epidemiology, Department of Biomedical Data Sciences, Leiden University Medical Center, Leiden, the Netherlands

**Keywords:** Illumina 450k arrays, DNA methylation, EWAS, RNA sequencing, Differential gene expression, TWAS, Statistical methods comparison

## Abstract

**Background:**

A large number of analysis strategies are available for DNA methylation (DNAm) array and RNA-seq datasets, but it is unclear which strategies are best to use. We compare commonly used strategies and report how they influence results in large cohort studies.

**Results:**

We tested the associations of DNAm and RNA expression with age, BMI, and smoking in four different cohorts (*n* = ~ 2900). By comparing strategies against the base model on the number and percentage of replicated CpGs for DNAm analyses or genes for RNA-seq analyses in a leave-one-out cohort replication approach, we find the choice of the normalization method and statistical test does not strongly influence the results for DNAm array data. However, adjusting for cell counts or hidden confounders substantially decreases the number of replicated CpGs for age and increases the number of replicated CpGs for BMI and smoking. For RNA-seq data, the choice of the normalization method, gene expression inclusion threshold, and statistical test does not strongly influence the results. Including five principal components or excluding correction of technical covariates or cell counts decreases the number of replicated genes.

**Conclusions:**

Results were not influenced by the normalization method or statistical test. However, the correction method for cell counts, technical covariates, principal components, and/or hidden confounders does influence the results.

## Background

Epigenomics and transcriptomics are important tools to investigate molecular mechanisms of disease etiology. Unlike the genome, the epigenome and transcriptome are dynamic and differ across tissues and over time [1–4]. Consequently, an epigenome-wide or transcriptome-wide association study (EWAS or TWAS, respectively) is influenced by more biological and technical factors than a genome-wide association study (GWAS). As a result, EWAS and TWAS methods are less standardized and do not always present the same results. For example, EWASs comparing current smokers with never smokers resulted in different significant CpGs and different numbers of significant CpGs per study, independent of sample size [5–15]. Similarly, TWASs comparing current smokers with never smokers found different numbers of associated genes [16–19]. Although these studies took place in different populations, they also used different analytical strategies, which could explain part of the variation in results.

For DNA methylation (DNAm) array data, previous studies compared different normalization methods [20–24]. Wu et al. concluded that most normalization methods performed similarly in association analyses when there was a strong association between CpGs and the exposure of interest [20]. To investigate the performance of DNAm values, Du et al. compared the use of beta values with *M* values in two samples and concluded that *M* values had better statistical properties, whereas beta values were more biologically interpretable [25]. Furthermore, white blood cell (WBC) counts are often used as important confounder adjustments for EWASs in whole blood. Cell counts estimated using the Houseman method [26] are commonly used when measured cell counts are not available. However, since the Houseman method is based on only six reference individuals [27], thorough investigation of this method based on large-scale DNAm data is needed. Lastly, principal components (PCs), surrogate variables (SVs), or unobserved covariates (also known as hidden confounders (HCs)) are commonly used methods to adjust for unmeasured hidden (technical or biological) confounders. Estimation of HCs using CATE has been suggested to outperform covariate adjustment using PCs or SVs [27, 28].

For RNA sequencing (RNA-seq) data, Li et al. compared a range of normalization methods and concluded that the commonly used options (e.g., DESeq/edgeR) provided the highest accuracy at the cost of decreased sensitivity compared to options with more specific applications [29]. When sufficient replicates (*n* > 4) per group were used, all methods performed similarly. Li et al. also compared normalization methods and concluded that commonly used options performed similarly, although some specific methods performed better for short (35 bp) read lengths and/or when alignment quality was low [29]. Several studies focused on other aspects of the analysis procedure such as the gene database used for quantifications (i.e., RefSeq, UCSC, and Ensembl) or sequencing platform and flowcell effect on results [30–32]. However, a comprehensive examination of multiple steps and combinations of analysis options is still lacking.

Most of these previous studies focused on a specific aspect of the procedure using simulated data or small datasets. To provide a complete evaluation of analysis strategies, we analyzed, replicated, and compared analysis strategies composed of commonly used normalization, correction, and association options in four large population-based datasets of the BIOS project, which have both DNAm array and RNA-seq data available [33, 34]. Because of this design, we can replicate results across cohorts and evaluate analysis strategies based on their replication performance. Our evaluation will help researchers select the optimal strategy and reduce unnecessary variation across studies. In addition, information about strategy differences will be helpful when comparing studies where different analysis strategies are used.

## Results

**Fig. 1:**
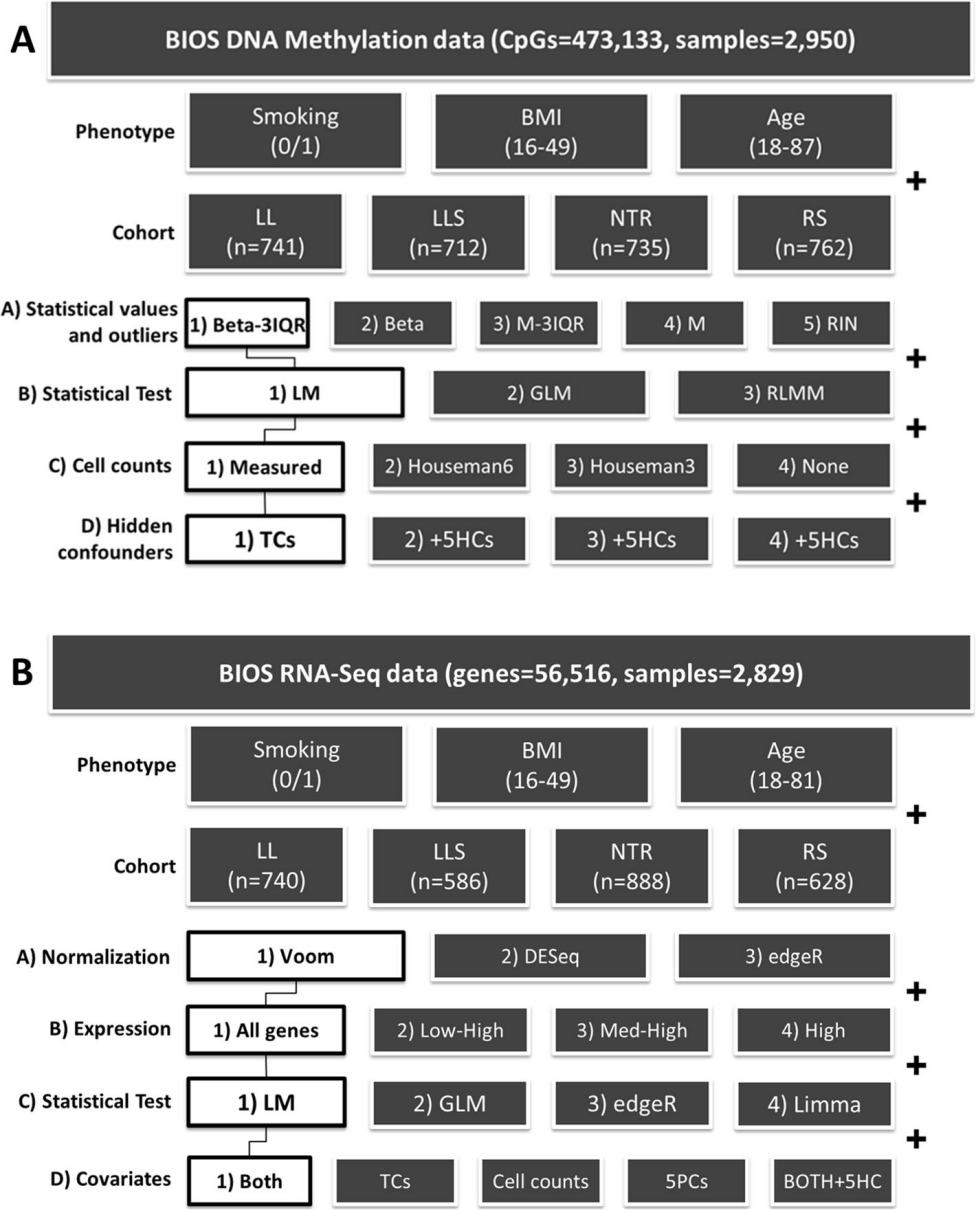
**a** Overview of DNA methylation analysis steps and commonly used options. We identified four steps in the procedure which often vary in literature: (A) DNAm value preprocessing, (B) statistical test, (C) cell count correction, (D) hidden confounder correction. We selected one combination of options and then varied these a single step at the time. These models were applied to age, BMI, and smoking. Each model was meta-analyzed in each combination of three discovery and one replication cohorts. The average replication rate and number of replicated genes of these four analyses were used to evaluate strategies. The base model is connected by the black line and includes Beta-3IQR dataset, an LM model, measured cell count correction, known technical confounder correction (TCs) (plate and row) and applying Bonferroni correction. HCs, hidden confounders, calculated after regressing out technical covariates (2), cell counts (3) or both (4). **b** Overview of gene expression analysis steps and commonly used options. We identified four steps in the procedure which often vary in literature: (A) normalization, (B) expression, (C) tests, and (D) technical covariates. We selected one combination of options and then varied these a single step at the time. These models were applied to age, BMI, and smoking. Each model was meta-analyzed in each combination of three discovery and one replication cohorts. The average replication rate and number of replicated genes of these four analyses were used to evaluate strategies. The base model is connected by the black line; Voom normalization, including all genes, a LM for statistical analysis, including technical covariates and cell counts and applying Bonferroni correction

Table 1 shows phenotypic characteristics for the four cohorts analyzed. To accommodate the differences in characteristics of the cohorts, cohorts were meta-analyzed. Figure 1 shows the various analysis strategies under evaluation. We selected a base model for DNAm and RNA-seq analysis comprised of one option in each category. Then, per category, we swapped the option in the base model with the alternatives and evaluate the replication performance against the base model. The categories for DNAm were (A) DNAm value preprocessing, (B) statistical test, (C) cell counts, and (D) hidden confounders. The categories for RNA-seq were (A) normalization method, (B) expression inclusion threshold, (C) statistical test, and (D) technical covariates.

**Table 1:** Characteristics of the four main cohorts at the time of blood draw. All entries represent averages with standard deviations unless otherwise indicated

	Phenotypes				DNA methylation				RNA-seq			
	LL	LLS	NTR	RS	LL	LLS	NTR	RS	LL	LLS	NTR	RS
	*n* = 761	*n* = 790	*n* = 1866	*n* = 768	*n* = 741	*n* = 712	*n* = 735	*n* = 762	*n* = 740	*n* = 579	*n* = 882	*n* = 628
Age	45 ± 13	58 ± 8	37 ± 14	68 ± 6	46 ± 13	59 ± 7	40 ± 15	68 ± 7	45 ± 13	59 ± 7	38 ± 15	69 ± 6
Sex (% male)	0.42	0.48	0.33	0.43	0.42	0.48	0.35	0.43	0.42	0.47	0.35	0.43
Smoking (% current)	0.15	0.11	0.19	0.10	0.15	0.12	0.19	0.10	0.15	0.13	0.18	0.09
BMI	25 ± 4	25 ± 4	24 ± 4	28 ± 4	25 ± 4	25 ± 3	25 ± 4	28 ± 4	25 ± 4	25 ± 3	25 ± 4	28 ± 4
Lymp (% of cells)	34 ± 8	29 ± 7	35 ± 9	36 ± 8	35 ± 7	29 ± 7	35 ± 9	36 ± 8	34 ± 8	29 ± 7	35 ± 9	36 ± 8
Mono (% of cells)	9 ± 2	5 ± 2	8 ± 3	7 ± 2	9 ± 2	6 ± 2	8 ± 3	7 ± 2	9 ± 2	6 ± 2	9 ± 3	7 ± 2
Gran (% of cells)	57 ± 8	63 ± 7	56 ± 9	57 ± 8	57 ± 8	63 ± 7	57 ± 9	57 ± 9	57 ± 8	63 ± 7	56 ± 9	57 ± 8

Each analysis strategy was meta-analyzed across three cohorts and replicated in the fourth, in all four combinations (the so-called leave-one-out method). Both meta-analysis and replication were defined by Bonferroni correction (*p* < 0.05) for the number of CpGs/genes tested. Below, we first describe the performance of the base model for methylation and expression data. Then, we describe, per category, how the various options affected the number of replicated signals (as a measure of sensitivity) and percentage of replicated signals (as a measure of true-positive rate in the discovery) and the overlap of significant CpGs/genes between analysis strategies. All results are Bonferroni corrected.

### DNA methylation strategy performance

**Fig. 2:**
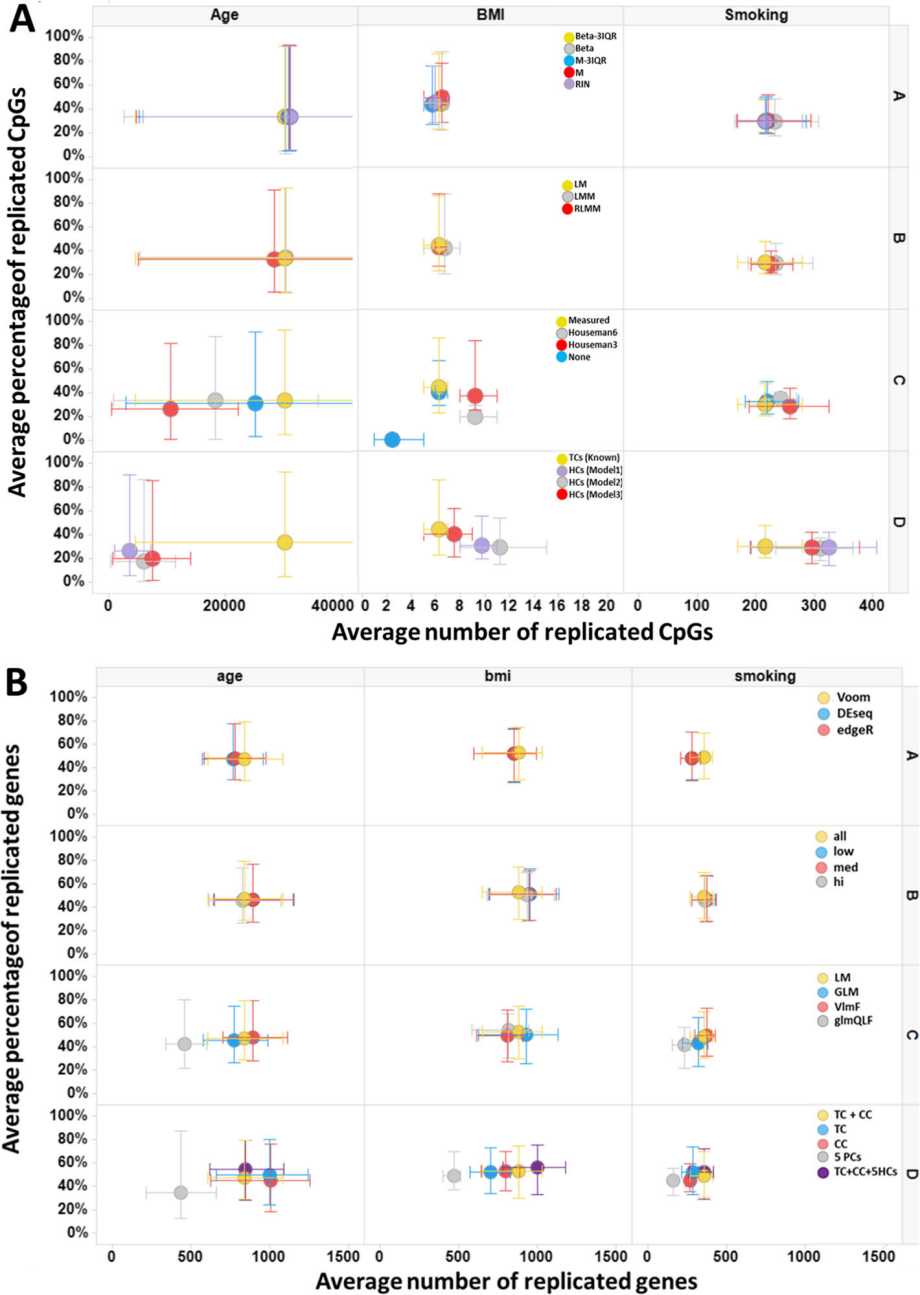
**a** The number (*x*-axis) and percentage (*y*-axis) of replicated CpGs for age, BMI, and smoking (shown in columns). Per row, each step of the analysis strategy is displayed. The yellow model is the reference model and remains the same in each column and row: Beta-3IQR dataset, standard linear model (LM), measured cell count correction, and known technical confounders (bisulfite conversion plate and array row) correction (TCs). The circles are average Bonferroni-corrected replication results. The bars indicate the range of the four leave-one-out analyses. In each row, the other (non-yellow) colors represent alternative options: (A) Datatypes: beta without exclusion of outliers in green, *M* values in red, *M* values with outlier exclusion using the 3IQR method in blue, and RIN in purple. (B) Statistical models: linear mixed models (LMM) in green and robust linear mixed models (RLMM) in red. (C) Cell count adjustment: Houseman6 in green, Houseman3 in red, and none in blue (see the “Methods” section for details). (D) Hidden confounder (HC) correction: model 1 in purple, model 2 in green, and model 3 in red (see the “Methods” section for details). **b** The number (*x*-axis) and percentage (*y*-axis) of replicated genes for age, BMI, and smoking (shown in columns). Per row, each step of the analysis strategy is displayed. The yellow model is the reference model and remains the same in each column and row: Voom normalization, including all genes, standard linear model (LM), correcting for technical covariates (TC) and cell counts (CC). The circles are average Bonferroni-corrected replication results. The bars indicate the range of the four leave-one-out analyses. In each row, the other (non-yellow) colors represent alternative options: (A) Normalization methods: DESeq normalization in blue and edgeR in red. (B) Gene inclusion: removing very low-expressed genes (blue), low-expressed genes (red), or medium-expressed genes (green). (C) Statistical models: A limma linear model Fit in red (limma), a standard GLM in blue, and the edgeR GLM adaptation in green. (D) Covariates: correcting solely for technical covariates (TC; blue) or cell counts (CC; red) or replacing both for the first five principal components (5PCs; green); the last option is by adding five hidden confounders (HCs) to the technical covariates and cell counts (5HCs; purple)

**Fig. 3:**
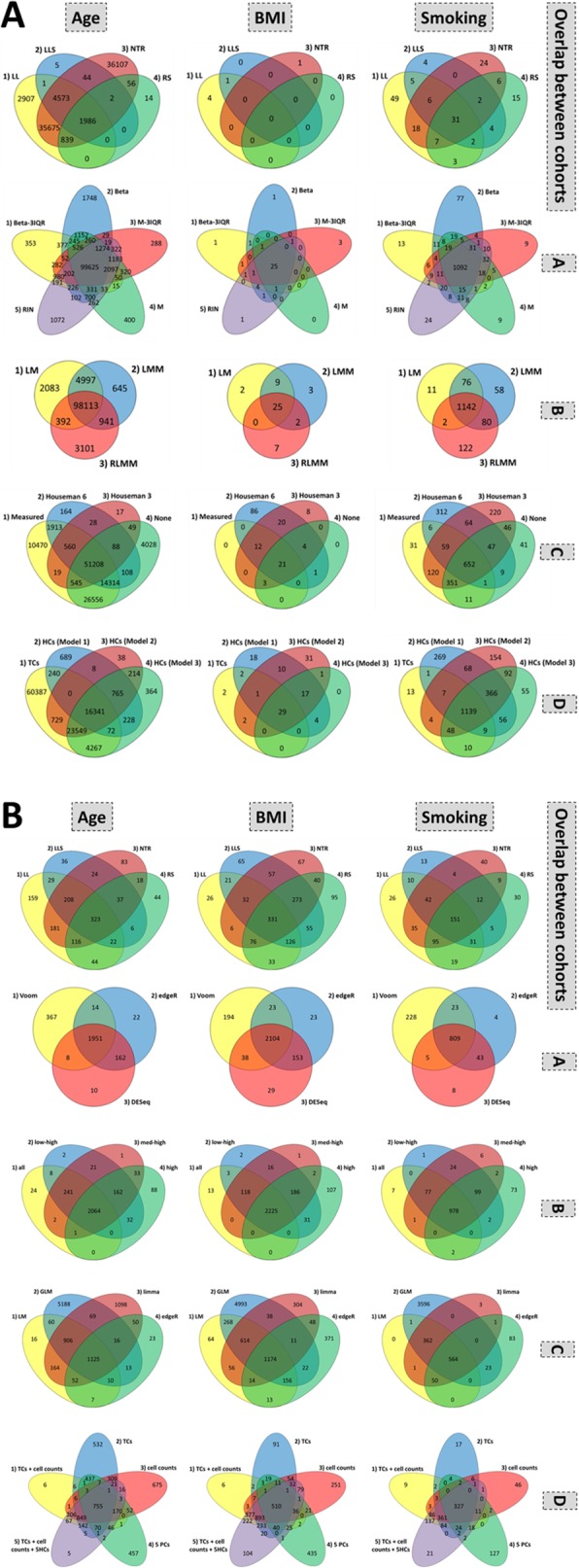
**a** CpG overlaps. The three 4-way Venn diagrams on top indicate the overlap in CpGs for each of the individual cohorts. These are based on the base model, using Bonferroni correction. The four diagrams below indicate the overlap between the strategies for each step, shown here for age, BMI, and smoking. These are the same strategies as shown in Fig. 2a. Yellow always represents the base model, and the green, red, blue, and purple colors belong to alternative strategies. (A) Beta values dataset in green, M-3IQR in blue, *M* in red, and RIN in purple. (B) LMM in green and RLMM in red. (C) Houseman6 imputed cell counts in green, Houseman3 imputed cell counts in red, and no cell count correction in blue. (D) Hidden confounder (HC) correction: model 1 (HCs independent of the exposure of interest, age, sex, known technical covariates, but not measured differential cell counts) in purple, model 2 (HCs independent of the exposure of interest, age, sex, measured differential cell counts, but not known technical covariates) in green, and model 3 (independent of the exposure of interest, age, sex, known technical covariates, and measured differential cell counts) in red. **b** Gene overlaps. The three 4-way Venn diagrams on top indicate the overlap in genes for each of the individual cohorts. These are based on the base model, using Bonferroni correction. The four diagrams below indicate the overlap between the strategies for each step, shown here for age, BMI, and smoking. These are the same strategies as shown in Fig. 2b. Yellow always represents the base model, and the blue, green, and red colors belong to alternative strategies. (A) DESeq normalization in blue and edgeR in red. (B) Removing very low-expressed genes (blue), low-expressed genes (red), or medium-expressed genes (green). (C) A limma linear model Fit in red, a standard GLM in blue, and the edgeR GLM adaptation in green. (D) Correcting for only technical covariates (blue) and only cell counts (red), adding five hidden confounders (purple), or replacing both for the first five principal components (green)

The base model included using normalized beta values and removing outliers based on the three interquartile range strategy (beta-3IQR), a linear model (LM), measured cell counts, and technical covariates, as described in more detail in the methods. This resulted in an average of 30,275 significantly replicated CpGs for age (range 4621–59,087), 6 replicated CpGs for BMI (range 5–7), and 217 replicated CpGs for smoking (range 168–279). The corresponding replication rates were on average 40% for age (range 5–93%), 52% for BMI (range 23–86%), and 31% for smoking (range 20–47%). All summary results are shown in Figs. 2a and 3a and Additional file 1: Table S1a. Below, we describe per category how different options influenced these results.

A) *DNAm value preprocessing:* For age, all normalization methods showed similar replication rates and slightly higher replication number compared to the base model. The same was observed for smoking, except that the RIN method performed more similar to the base model than the beta, *M*, or M-3IQR methods. The replicated number and rate of CpGs were largely the same across methods. For BMI, given the small numbers of CpGs (e.g., 6 for the base model), it was difficult to robustly compare results.
B) *Statistical tests:* Compared to the base model, a linear mixed model (LMM) reported a slightly higher number of replicated hits for age and smoking. The robust linear mixed model (RLMM) reported lower numbers of replicated CpGs for age and similar number of replicated CpGs for smoking. Replication rates were nearly identical to the LM base model for all exposures. The replicated CpGs were shared across methods.
C) *Cell count adjustment:* Without correction for cell counts, fewer replicated CpGs were found for age (83% compared to the number of replicated CpGs in the base model), but no differences were seen for BMI and smoking (Fig. 2a). For age, adjusting for Houseman imputed cell counts substantially decreased the number of significantly replicated CpGs; Houseman6 resulted in 18,368 CpGs for age (61% of the base model), and Houseman3 resulted in 10,678 CpGs for age (35% of the CPGs compared to the base model). The replication rate with Houseman6 was similar as compared to the base model, but Houseman3 resulted in a slightly lower replication rate as compared to the base model. For smoking, using Houseman imputed cell counts resulted in a slightly higher number of replicated CpGs; Houseman6 resulted in 243 CpGs (112% compared to the base model), while Houseman3 resulted in 259 CpGs (119% compared to the base model). When examining the overlap between the CpGs in the different cell count adjustment strategies across all four cohorts (Fig. 3a) for smoking, we observed that a total of 652 CpGs were common for all cell count adjustment methods. In addition, a relatively large number of CpGs were only observed by Houseman6 and 3, respectively (312 and 220 CpGs).
D) *Correction for hidden confounders (HCs):* HCs were calculated in three additional models (model 1 being the base model); model 2, HCs independent of the described covariates, but not measured differential cell counts; model 3, HCs independent of the described covariates, but not known technical covariates; and model 4, using HCs independent of the exposure of interest, age, sex, known technical covariates, and measured differential cell counts. For age, adjusting for five HCs resulted in a decreased number of significantly replicated CpGs: 7509 in model 4 (25% compared to the base model), 6054 in model 3 (20% compared to the base model), and 3621 in model 2 (12% compared to the base model). In contrast, for BMI and smoking, these three HC models showed an increase in the number of significantly replicated CpGs: 8, 9, and 10 for BMI and 297 (137% of the base model), 311 (143% of the base model), and 325 (150% of the base model) for smoking in models 4, 3, and 2, respectively. Thus, for age, a large number of CpGs were not detected when correcting for HCs, while for smoking and BMI, a number of CpGs were found only when using HC correction. The replication rates were very similar across all models.

### RNA sequencing strategy performance

The base model (Voom normalization, no expression inclusion threshold, LM, technical covariates, and measured cell counts) resulted on average in 842 significantly replicated genes for age (range 610–1082), 881 replicated genes for BMI (range 651–1029), and 354 replicated genes for smoking (range 268–409). The corresponding mean replication rates were 54% for age (range 28–80%), 55% for BMI (range 30–74%), and 51% for smoking (range 30–69%). Below, we describe per category how different options influenced these results, as available in Additional file 1: Table S1b and shown in Figs. 2b and 3b.

A) *Normalization method:* The DESeq and edgeR normalization methods reported a slightly lower number of replicated genes with the same replication rate compared to the base model (93% and 91% of the base model, respectively). The normalization method did not influence which genes were replicated. This pattern was observed for all three exposures.
B) *Gene expression inclusion criteria:* Including low (average CPM > 1 in 20% of samples) and higher expressed genes (1. low) or medium (average CPM > 1) and higher expressed genes (2. med) provided slightly more replicated genes for age (both 107% compared to the base model) at a similar replication rate. The most stringent threshold (3. hi) also resulted in a similar replication number (98% compared to the base model) and percentage (98% compared to the base model). Mostly the same genes were replicated regardless of the inclusion threshold.
C) *Statistical tests:* limma’s linear model fit (limma) test resulted in slightly more replicated genes, at the cost of a lower replication rate (lower specificity). The glmQLF test from edgeR showed a lower number of replicated genes. GLM showed nearly the same results as the base model. These findings were consistent across the exposures, with smaller differences for BMI.
D) *Covariates:* For age, correcting solely for technical covariates or cell counts resulted in a large increase (119% compared to the base model) in replicated genes. For BMI and smoking, the number of replicated genes, as well as the replication rate, decreased when removing these covariates. Correcting for five principal components instead of technical covariates or cell counts decreased the number of replicated signals to 51%, 53%, and 46% of the base model for age, BMI, and smoking, respectively. Similarly, the replication rate decreased to 87%, 96%, and 96% for age, BMI, and smoking compared to the base model, respectively. Conversely, five hidden confounders added to the technical covariates and cell counts in the base model increased the replication number to 100.4%, 114%, and 101.4% compared to the base model for age, BMI, and smoking, and increased the replication rate to 107%, 103%, and 103% of the base model for age, BMI, and smoking, respectively. In addition to finding fewer replicated genes after PC correction, the identified genes were not the same as the base model, and other methods did not observe these genes. Similarly, when adding five HCs, many genes identified in the model with HCs were not observed in the other models, but the difference was smaller than that for the model including PCs.

### FDR instead of Bonferroni correction

In addition to the comparisons described above, all analyses were also repeated using FDR correction in the discovery analysis instead of Bonferroni correction. All analyses using FDR showed a higher number of replicated CpGs and genes, at the cost of a much smaller replication rate. For example, for the base model for age, 30,275 CpGs and 842 genes were replicated at replication rates of 40% and 47%, respectively, when using Bonferroni correction. When using FDR correction, the number of CpGs increased by 18% and the replication rate decreased by 18%. Similarly, the number of genes increased by 98% and the replication rate decreased by 20%.

### METAL or GWAMA for meta-analysis

As the GWAMA tool requires input that is not provided by some RNA expression statistical methods, we opted to use only METAL for the RNA-seq analysis. For those RNA-seq models where both could be run, the results were identical.

### Evaluation using different *p* value cutoffs

The results for additional *p* value cutoffs (FDR, uncorrected < 1 × 10^–8^ and uncorrected < 0.05) are available in Additional file 1: Table S1 and Additional file 2: Figure S1. Less stringent cutoffs led to an increase in absolute numbers of replicated signals but at a decreased relative replication rate for both DNAm and RNA-seq. Most models responded similarly to this change, and the respective performance between methods did not change.

For BMI and smoking in the DNAm analyses, the lowest threshold *p* < 0.05 showed fewer replicated CpGs as compared to the other three thresholds. This was caused by a 333-fold increase of significant CpGs in discovery meta-analysis for BMI and an 8.6-fold increase for smoking when we used the lowest threshold in comparison to the FDR threshold. In contrast, the discovery meta-analysis showed only a 1.12-fold increase of significant CpGs for age. As a result, the Bonferroni threshold for replication was strongly increased, and most of the previously replicated CpGs did not survive this threshold.

For the normalization options (A) and covariate correction options (D) in RNA-seq analyses, the respective differences between the options were unchanged depending on the *p* value cutoff. For the gene inclusion thresholds (B), it showed that including only the most highly expressed genes yields a slightly higher replication rate using the uncorrected *p* value threshold. For the statistical test comparison (C), using lower *p* value thresholds (FDR and uncorrected) provided a more pronounced difference between the models.

### Categorical analyses for age and BMI

For DNAm and RNA-seq, when we used age/BMI as categorical instead of continuous exposures, the differences between methods remained largely the same. However, the categorical models consistently resulted in a lower number and percentage of significantly replicated CpGs/genes as compared to the continuous models. The only exception was in the hidden confounder (HC) correction model for age, where the categorical models resulted in a larger number of significantly replicated CpGs/genes as compared to the continuous models. The results for these categorical models can be found in Additional file 1: Table S1 and Additional file 3: Figure S2.

## Discussion

We evaluated commonly used analysis strategies for population-based datasets for DNA methylation and RNA sequencing in almost 3000 participants from four Dutch cohorts. For each step in the analysis procedure, we compared commonly used options and reported their influence on the exposure of interest. These results will aid in comparing studies with different analysis strategies and can help in the choice between alternative analysis strategies.

The four included cohorts differed on some important parameters (e.g., age). As a combined dataset would not have easily been able to distinguish true age effects from batch effects between age-differing cohorts, we decided to run cohort-level analyses first and then meta-analyze the datasets, as is commonly done in meta-analyses of “omics” data [35]. As these exposure differences will also result in different power between cohorts for each exposure, we meta-analyzed each combination of three cohorts and replicated in the fourth [36]. Therefore, when a cohort of low power for an exposure performs poorly as replication cohort, while a powerful cohort for that exposure replicated many signals, these effects were averaged out and provided a reasonable aggregated performance of each strategy [37].

For DNA methylation data, our evaluation leads to the following considerations/recommendations:

*DNAm value preprocessing:* There were no large differences between the different methylation values. We suggest to use beta-3IQR in order to avoid spurious findings based on DNA methylation outliers, but we do not expect another option to have a large influence on the results.

*Statistical tests:* The theoretical advantage of using an RLMM over LM or LMM is considered to be that it is less sensitive to exposure and methylation outliers and heteroscedasticity. However, LM, LMM, and RLMM provided nearly identical results, and the analysis running time for RLMM is considerably longer. Therefore, LM or LMM approaches might be preferred as they are simple and widely used base-R functions.

*Cell count adjustment:* Beforehand, we expected that differential cell counts are a major influence on DNA methylation data measured from whole blood [38]. Indeed, we observed a large influence of cell counts on age, but not on BMI or smoking. These results were in line with previous work which also found that adjusting or not adjusting for blood cell counts had no substantial impact on EWASs of BMI and smoking [39]. For all exposures, we observed influence of Houseman6/3 cell counts on the analysis, with a larger deviation from the measured cell counts (base model) for Houseman3 than Houseman6. Therefore, we recommend the adjustment for measured cell counts if available. If not, the Houseman6 estimated six cell counts could be used for exposures other than age.

*Correction for HCs:* Adjusting for five HCs substantially influenced the results. For age, adjusting for five HCs substantially decreased the number of replicated CpGs. For BMI and smoking, adjusting for five HCs seemed to improve the results by improving the number of replicated CpGs. Therefore, for exposures other than age, adjusting for HCs is highly recommended in order to remove unknown variation from the data.

For RNA expression data, our evaluation leads to the following considerations/recommendations:

*Normalization method:* There was no large influence of normalization methods. The Voom method resulted in slightly more replicated genes and is recommended.

*Gene expression inclusion threshold:* The gene inclusion threshold displayed minimal influence on the results. To be complete, it is suggested to include and report all genes in the dataset.

*Statistical method:* In our datasets, the standard LM/GLM models performed similarly to the custom limma/edgeR methods. However, it is possible that datasets of smaller sample sizes (e.g., fewer than 20 samples) benefit more from the custom methods. For larger datasets, the standard, widely used LM and GLM are easier to use and could provide easier compatibility with other applications (e.g., meta-analysis).

*Covariates:* In our results, correcting for PCs did not improve performance and is not recommended when technical covariates and/or cell counts are available. In our datasets, the PCs correlated to the technical covariates, to the cell counts, and in some occasions to the exposures (mostly age); this likely led to overcorrection when PCs where added on top of these covariates. Correcting for five hidden confounders on top of the base model improved the results for all exposures and is recommended to use. When doing so, care should be taken that the hidden confounders are not correlated to the exposure of interest (or a confounder which is correlated to the exposure) which could remove true results. At current, adjusting for confounders using HCs is not the standard practice in RNA-seq analysis, but should be implemented more widely based on these findings. Additionally, we did not use the Bacon package to correct for inflation of test statistics, as this is not yet widely used for RNA-seq data. However, applying bacon correction on RNA-seq data is becoming more common and should be considered in future RNA-seq studies [28].

### Evaluation using different *p* value cutoffs

For all models, we observed a balance with more stringent *p* value cutoffs resulting in fewer replicated signals, but a larger replication rate. In general, we recommend using Bonferroni-corrected *p* values with a cutoff of *p* < 0.05. The FDR-corrected *p* values can provide an alternative. Decreasing the *p* value threshold stringency always leads to increased false positives and thus a lower replication rate. Using uncorrected *p* value cutoffs (whether nominal 0.05 or a too conservative 1E−8) is not recommended.

For DNAm, the differences between methods were similar for all thresholds, and the main conclusions did not change. For RNA-seq, these results further show that the GLM and edgeR’s glmQLF models are more conservative (lower number but higher percentage of replicated signals) while limma’s linear model fit is more liberal (higher number but lower percentage of replicated signals) compared to the base model. The LM model is still recommended.

### Categorical analyses for age and BMI

To assess whether strategies are influenced by the continuous or categorical definition of the exposure, we analyzed age and BMI both as continuous and categorical (i.e., highest versus lowest tertiles) exposures of interest. All models responded similarly to the categorical exposure in comparison to the continuous exposure, showing lower number and percentage of replicated signals, indicating lower power for categorical exposures. For both DNAm and RNA-seq analyses, we observed differences in performance between models only with HC correction. The models with five HCs for age performed worse when we used age as a categorical variable with the highest vs lowest tertiles and excluded the middle tertile. Likely, these results indicate that HCs are insufficiently adjusted for age when it is included as a categorical variable (compared to continuous). Overall, these results seem robust for categorical/continuous exposure definitions, but do emphasize that HC correction may be challenging when working with categorical exposures. For continuous variables and most categorical variables (e.g., BMI tertiles and smoking), using HCs performed best and is still recommended.

Although most of the differences we observed between strategies were consistent across exposures and cohorts, these results might not be applicable to all other DNAm array or RNA-seq studies. For example, we have studied three exposures for which we could observe relatively large differences in blood methylation or expression, with the exception of BMI in methylation. We observed differences in performance between exposures, for example, when correcting for different cell counts, HCs or PCs in age, or the low number of replicated CpGs for BMI. As such, a universally optimal model could not be defined and performance of these different strategies needs to be confirmed for other exposures. However, performance differences between many strategies were consistent across exposures (specifically BMI and smoking), individual cohorts and DNAm/RNA-seq datasets, and will likely hold even in other exposures or datasets.

In this study, we have compared multiple analysis strategies on four cohorts and suggested a base model to reduce heterogeneity between studies. The most ideal validation would be to re-analyze a number of published studies using this optimal model and demonstrate a decrease in heterogeneity between results of previous analyses and those with the new model. However, to our knowledge, for none of the studies we investigated this was possible, due to lack of publically available phenotypic information or lack of publically available individual-level DNAm/RNA-seq data. As it may not always be possible to share such data publicly, this further shows the need for more standardized DNAm/RNA-seq methods, so results between studies can be compared more easily.

Similarly, we studied four relatively large population-based studies. Results obtained from smaller studies, or other types of populations, for example, patients or samples of extreme exposures, might yield different results and require alternative strategies. These comparisons were beyond the scope of our study, which focused on commonly used strategies. Our results might be most generalizable to population-based DNAm and RNA-seq studies. Finally, our study lacked a gold standard, which will have limited our ability to distinguish strategies with many false positives from strategies with a high sensitivity. Despite these factors, we evaluated the consistent influences of analysis strategies and options and reported analysis suggestions for both datatypes. We hope that these results will aid other researchers in selecting an appropriate analysis strategy and/or in evaluating the impact, a certain strategy might have had on the observed results.

## Conclusions

Based on our findings, for DNA methylation studies, we recommend to correct for measured cell counts when available and include additional hidden confounders (independent of cell counts and technical covariates) in the statistical model. We suggest using Beta-3IQR values and the LM statistical test for DNAm studies, although alternatives will yield similar results and can also be used. For RNA sequencing studies, we recommend using hidden confounders in addition to technical covariates and measured cell counts. The use of principal components is not recommended. We recommend using the Voom normalization method and suggest to include all genes in the analysis (independent of expression level). Finally, we suggest using a LM or GLM statistical model for large studies and a custom method like limma/edgeR for smaller studies. Our results show a large difference in replication results between cohorts, and therefore, using replication in DNAm or RNA-seq analysis is also recommended.

## Methods

### Data generation

Generation of the BIOS gene expression dataset was described previously [33, 34]. In short, DNA and RNA were collected from 3296 unrelated participants of six Dutch populations as described below. Analyses were restricted to four large cohorts; LifeLines (LL), Leiden Longevity Study (LLS), Netherlands Twin Register (NTR), and Rotterdam Study (RS). We included 2950 participants with DNAm array data and 2829 participants with RNA-seq data. Characteristics for these cohorts are described in Table 1.

#### DNA methylation data

Whole blood was used to isolate genomic DNA. Five hundred nanograms of genomic DNA was bisulfite converted using the EZ DNA Methylation kit (Zymo Research, Irvine, CA, USA). Methylation profiling was then performed using Infinium Illumina HumanMethylation 450k arrays according to the manufacturer’s protocol. Quality control of the samples was performed using MethylAid [40]. Probes with either a high detection *p* value (> 0.01), low bead count (< 3 beads), or low success rate (missing in > 5% of the samples) were set to missing. Samples were excluded from the analysis if they contained an excess of missing probes (> 5%). Imputation was performed per cohort, subsequently, to impute the missing values [41]. The raw beta values were normalized using functional normalization [22] as implemented in the minfi package [42]. The normalized beta values were log_2_ transformed to produce *M* values [42].

#### RNA-seq data

Total RNA was derived from the whole blood, depleted of globin transcripts using Ambion GLOBINclear, and subsequently processed using the Illumina TruSeq v2 library preparation kit. On average, 40 million paired-end reads of 50 bp were generated per participant using illumina’s Hiseq 2000. Samples were demultiplexed using CASAVA and aligned to the hg19 reference genome using STAR [43]. Alignments were sorted, read groups were added using picard [44], and gene expression was quantified using featureCounts [45]. We selected participants for which all covariates were available (sex, age, BMI, smoking status, and measured cell counts). Raw count matrices per cohort were used for analysis.

### Base model and analysis

The main steps in epigenomic and transcriptomic analyses often vary between studies, as shown in Fig. 1a and b, respectively. First, we compiled a base model with a single option from each step in Fig. 1a and b. These options were then replaced, one at a time, in the various analysis strategies. These strategies were applied to three exposures of interest (age, BMI, and smoking status) in each cohort (LL, LLS, NTR, and RS). Every combination of three discovery cohorts was meta-analyzed and replicated in the remaining cohort (leave-one-out method). The average number and percentage of replicated CpGs/genes were calculated from these four results and were used to evaluate the performance of each strategy. Age, sex, measured percentages of WBC counts (granulocytes, lymphocytes, and monocytes), and technical covariates specified below were included as covariates unless specified otherwise. Replication analyses were always Bonferroni corrected. Meta-analyses were performed using GWAMA (DNAm array data) [46] or METAL (RNA-seq data) [47].

#### DNA methylation array-specific analysis strategies

The technical covariates used for each DNAm array analysis were bisulfite conversion plate and array row. All analyses were corrected for inflation and bias using the Bacon package [28], which estimates empirical null distribution using the Bayesian method. The following steps were investigated in detail (see Fig. 1a).

A) Methylation values: We investigated five types of DNAm values, namely (1) beta values, representing the percentage of methylation between 0 (unmethylated) and 1 (methylated) [25]; (2) beta-3IQR values, where beta values of outlier samples per methylation CpG were removed (replaced with NAs) using the three interquartile range (IQR) strategy, i.e., any beta value below quartile (Q)_1_ − 3×IQR or above Q_3_ + 3×IQR was removed [48]; (3) *M* values, calculated as the log_2_ ratio of the methylated probe intensity and unmethylated probe intensity [49]; (4) M-3IQR values, where *M* values of outlier samples per methylation CpG were removed using the 3xIQR strategy as described above [48]; and (5) RIN (rank-based inverse normal transformation) values, wherein beta values for each sample were ranked and replaced with the corresponding standard normal quantiles in order to create a normal distribution [50]. We selected beta-3IQR values for the base model.
B) Statistical tests: We investigated three types of linear models: (1) linear regression model (LM), (2) linear regression mixed model (LMM), and (3) robust linear regression mixed model (RLMM). We selected LM for the base model.
C) Cell count correction: (1) For the base model, we used the percentages of differential measured cell counts of granulocytes, lymphocytes, and monocytes. This base model was compared with three other models: (2) a model without cell count correction, (3) a model adjusted for the cell subtypes imputed with the reference-based Houseman method [26], using the default percentage counts of all six imputed cell types: granulocytes, monocytes, NK cells, B cells, CD4+, and CD8+ T lymphocytes. We refer to this as “Houseman6”, (4) a model adjusted for the same imputed cell counts, but using three instead of six cell types: granulocytes, monocytes, and lymphocytes (sum of NK cells, B cells, CD4+, and CD8+ T lymphocytes) in order to match with measured cell counts of the base model. We refer to this as “Houseman3.”
D) Hidden confounder (HC) correction; (1) For the base model, we used known technical confounder correction (bisulfite conversion plate and array row). This base model was compared with three more models that were corrected for HCs calculated from the CATE package [27, 28]. These were calculated per cohort per exposure. (2) We calculated five HCs independent of the exposure of interest (BMI or smoking), age, sex, and known technical covariates. However, we did not regress out measured differential cell counts, and therefore, we assume that the HCs reflect cell counts. This model contained age, sex, technical confounders, and five HCs as covariates. (3) HCs were calculated by regressing out the exposure of interest, age, sex, and also measured differential cell counts. In this case, we did not regress out known technical confounders, and therefore, these HCs are thought to reflect technical confounders. This model contained age, sex, measured differential cell counts and 5 HCs as covariates. (4) HCs were calculated by regressing out not only the exposure of interest, age, and sex, but also the measured differential cell counts and known technical covariates. In this case, HCs can be regarded as any more potential hidden biological or technical confounders that might influence the data in addition to the differential cell counts and technical confounders’ correction. This model contained age, sex, measured differential cell counts, known technical confounders, and five HCs as covariates.

#### RNA sequencing-specific analysis strategies

All RNA-seq strategies were corrected for technical covariates: sequencing batch (flow cell) and average GC percentage in the reads, in addition to the biological covariates mentioned before. We compared the following steps in detail (see also Fig. 1b).

A) Normalization method: Three commonly used RNA-seq normalization methods: (1) Voom, (2) edgeR, and (3) DESeq, were investigated. The edgeR and DESeq methods adopted a Trimmed mean of *M* value normalization (TMM) [51, 52]. Voom adopted edgeR’s normalization but first raised zeros to a minimum value of 1 and performed a log transformation [53]. We selected Voom for the base model.
B) Expression inclusion criteria: We varied the genes allotted to normalization using four common inclusion CPM (counts per million) thresholds of gene expression. (1) All genes expressed at any level in at least one sample were included. (2) All genes with a CPM ≥ 1 in ≥ 20% of the samples were included. (3) Genes with an average CPM ≥ 1 across all samples were included. (4) All genes with an average CPM ≥ 10 across all samples were included. In the base model**,** all genes were included (option 1).
C) Statistical tests: We used four commonly used statistical tests: (1) a default linear model (LM) [54]; (2) a default generalized linear model (GLM) with negative binomial distribution; (3) the linear model fitfunction of the limma package, which was a weighted linear model where genes with a large variance (e.g., genes with very low expression) had lower weights; (4) the edgeR’s generalized linear model fit (glmQLF), which used a negative binomial distribution followed by a log ratio likelihood (LR) test. Options 3 and 4 were RNA-seq-specific hierarchical models that take into account differences in variance estimates across genes [51, 53]. Option 1 was included in the base model. Option 4 was also run on the Voom normalized dataset. Option 2 and 3 were run on the edgeR normalized dataset as the negative binomial distribution did not apply after Voom’s log transformation.
D) Technical correction: We used five commonly used approaches to correct for technical factors. (1) We included technical covariates (GC percentage and flow cell) and measured cell counts. (2) Corrected only for technical covariates. (3) Corrected only for cell counts. (4) Replaced technical covariates and cell counts by the first five principal component PCs, calculated per cohort using the prcomp function in R. (5) Added five hidden confounders to the technical covariates and cell counts. Hidden confounders were calculated per cohort per exposure and were adjusted for the respective exposure, age, sex, technical covariates, and cell counts.

### Evaluating strategy performance

In each analysis, three of the four cohorts were meta-analyzed in the discovery and the fourth cohort was used for replication. We repeated for each combination of three discovery and one replication cohort. The number of significantly replicated CpGs/genes was obtained for each repetition, as well as the percentage of CpGs/genes from discovery that reached replication (replication rate). For both the number and percentage of replicated signals, the average of the four combinations was calculated and used to evaluate performance of each strategy. We compared each strategy to the base model and looked for consistent differences in replication number or percentage across exposures.

### Categorical analyses for age and BMI

In order to investigate whether an optimal analysis strategy is dependent on whether the independent variable is continuous or categorical, we expanded our association analyses on age and BMI by converting them into tertiles. We used the highest and lowest tertiles to define the categories. The results of these categorical analyses were compared with the results of the continuous analyses where age and BMI were used as continuous measures. For DNAm, we did not analyze BMI into categorical exposure because the numbers of significantly replicated CpGs were already small for the continuous models (average of < 12 CpGs) when a Bonferroni threshold was used for multiple testing. This made it difficult to draw conclusions when comparing different methods within continuous models and therefore would have made it even more difficult to compare results between categorical models.

### Evaluation using different *p* value cutoffs

For all the comparisons mentioned, both discovery and replication results were Bonferroni corrected. In addition to using the Bonferroni threshold for the discovery results, we applied three other thresholds to evaluate the robustness of the approaches: (1) Benjamini-Hochberg FDR threshold (FDR *p* value < 0.05), (2) highest threshold (uncorrected *p* value threshold < 1 × 10^–8^), and (3) lowest threshold (uncorrected *p* value threshold < 0.05). Differences between models were compared between *p* value thresholds to establish that the models show similar (respective) results independent of *p* value thresholds.

In addition, for each strategy, we performed a meta-analysis of all four cohorts for DNA methylation and RNA expression. Overlaps in CpGs/genes between all strategies per step were determined using Venn diagrams to ascertain if the same CpGs/genes were identified between strategies [55].

## Supplementary information

**Additional file 1: Table S1.** Results of all DNAm (A) and RNA-seq (B) models.

**Additional file 2: Figure S1.** Results of the alternative multiple testing corrections methods for the DNAm (A) and RNA-seq models (B).

**Additional file 3: Figure S3.** Results of the Age and BMI categorical analysis for DNAm (A) and RNA-seq (B) models.

**Additional file 4.** Review history. Comments of the reviewers and authors’ responses.

## Data Availability

The datasets from BIOS are available from the European Genome-Phenome Archive by accession number EGAS00001001077 (https://www.ebi.ac.uk/ega/studies/EGAS00001001077). Alternative options to access the data are available through the BIOS website; https://www.bbmri.nl/acquisition-use-analyze/bios/ [34].
