# Supplementary material for: Evaluation of commonly used analysis strategies for epigenome- and transcriptome-wide association studies through replication of large-scale population studies
Source: Genome Biol. 2019 Nov 14;20:235. doi: 10.1186/s13059-019-1878-x (PMC6857161; doi:10.1186/s13059-019-1878-x)
Supplement: Supplementary file 2 — Additional file 2: Figure S1. Results of the alternative multiple testing corrections methods for the DNAm (A) and RNA-seq models (B). [file 13059_2019_1878_MOESM2_ESM.docx]

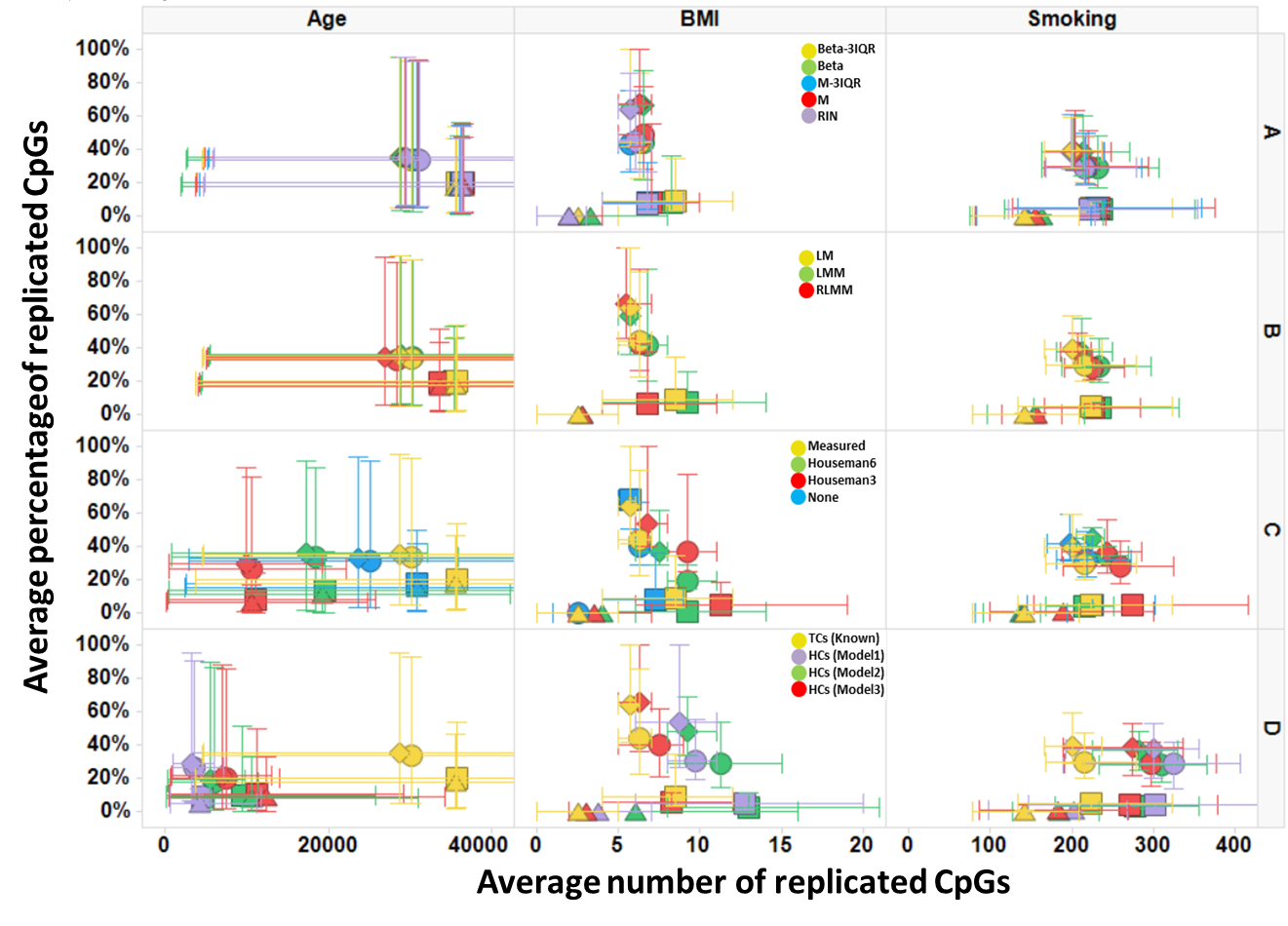


Fig S1a: The number (x-axis) and percentage (y-axis) of replicated CpGs for the DNAm p-value cutoff analyses for age, BMI and smoking. Per row, each step of the analysis strategy is displayed. The yellow model is the reference model and remains the same in each column and row: Beta-3IQR dataset, standard linear model (LM), measured cell counts correction and known technical confounders (bisulfite conversion plate and array row) correction (TCs). The shapes; (1) Circles are previous Bonferroni threshold (corrected p-value <0.05) (2) Squares are Benjamini-Hochberg FDR threshold (corrected p-value <0.05) (2) Diamonds are highest threshold (uncorrected p-value threshold <1x10^-8^) and (3) triangles are lowest threshold (uncorrected p-value threshold <0.05). The bars indicate the range of the four leave-one-out analyses. In each row, the other (non-yellow) colors represent alternative options: A) Datatypes: Beta without exclusion of outliers in green, M-values in red, M-values with outlier exclusion using the 3IQR method in blue and RIN in purple. B) Statistical models: linear mixed models (LMM) in green and robust linear mixed models (RLMM) in red. C) Cell count adjustment: Houseman6 in green, Houseman3 in red and none in blue (see methods for details) D) Hidden confounders (HCs) correction; Model 1 in purple, Model 2 in green and Model 3 in red (see methods for details).


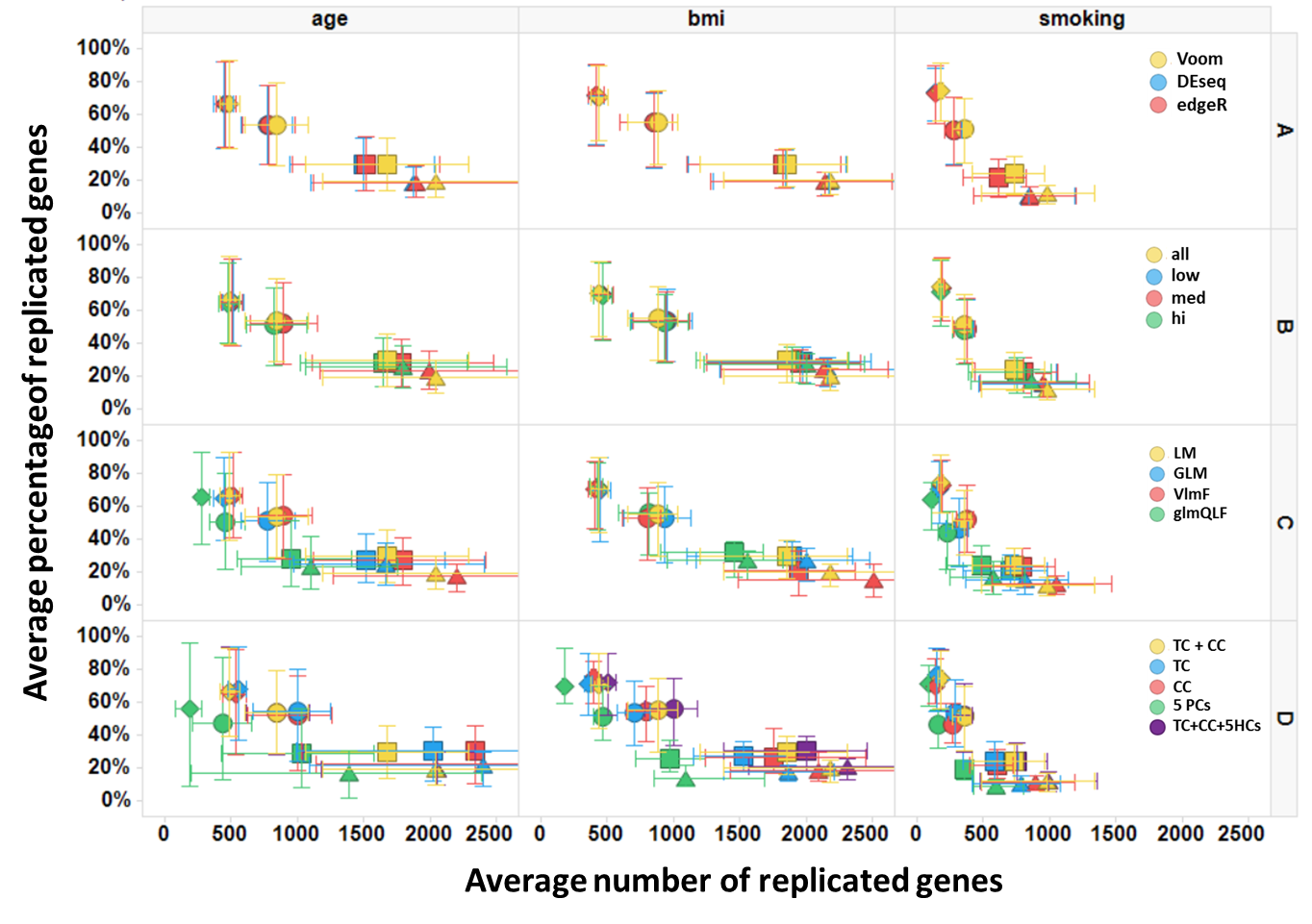


Fig S1b: The number (x-axis) and percentage (y-axis) of replicated genes for the RNA-seq p-value cutoff analyses for age, BMI and smoking. Per row, each step of the analysis strategy is displayed. The yellow model is the reference model and remains the same in each column and row: Voom normalization, including all genes, standard linear model (LM), correcting for technical covariates (TC) and cell counts (CC). The shapes; (1) Circles are previous Bonferroni threshold (corrected p-value <0.05) (2) Squares are Benjamini-Hochberg FDR threshold (corrected p-value <0.05) (2) Diamonds are highest threshold (uncorrected p-value threshold <1x10^-8^) and (3) triangles are lowest threshold (uncorrected p-value threshold <0.05). The bars indicate the range of the four leave-one-out analyses. In each row, the other (non-yellow) colors represent alternative options: A) Normalization methods: DESeq normalization in blue and edgeR in red. B) gene inclusion: removing very low-expressed genes (blue), low-expressed genes (red) or medium-expressed genes (green). C) Statistical models: A limma linear model Fit in red (limma), a standard GLM in blue and the edgeR GLM adaptation in green. D) Covariates: correcting solely for technical covariates (TC; blue) or cell-counts (CC; red) or replacing both for the first 5 principal components (5PCs; green), the last option is by adding 5 hidden confounders (HCs) to the technical covariates and cell counts (5HCs; purple).
