# Supplementary material for: Evaluation of commonly used analysis strategies for epigenome- and transcriptome-wide association studies through replication of large-scale population studies
Source: Genome Biol. 2019 Nov 14;20:235. doi: 10.1186/s13059-019-1878-x (PMC6857161; doi:10.1186/s13059-019-1878-x)
Supplement: Supplementary file 4 — Additional file 4. Review history. Comments of the reviewers and authors’ responses. [file 13059_2019_1878_MOESM4_ESM.docx]

Review History

**First round of review**

**Reviewer 1**

**Are you able to assess all statistics in the manuscript, including the appropriateness of statistical tests used?**

Yes, and I have assessed the statistics in my report.

**Comments to author:**

In this manuscript, the authors evaluated the commonly used analysis strategies for EWAS and TWAS studies, and they tried to give practical guidelines for choosing suitable strategies. I do acknowledge the values of such evaluation studies. However, the value of this study is limited due to the following three major considerations.

1.     The practical guidelines provided in this study are based on the analysis of four cohorts. Those guidelines are valuable only if they can be applied to other datasets. For example, the smoking / non-smoking datasets mentioned in background (ref 16-19). The authors mentioned that using different analytical strategies can partially explain the variation between results from different cohorts. To prove the usefulness of the practical guidelines, the authors could examine whether using the recommended strategies of this study to re-analyze those public datasets can significantly reduce the variation. 

2.     This study evaluated the optimal analysis strategies for age, BMI and smoking, and obtained distinct strategies. The authors should investigate the causes of such phenotype-dependent optimal strategies. For example, smoking is a binary variable, while BMI is a continuous variable. Whether a strategy is specifically suitable for binary or continuous variables? Why? 

3.     In this manuscript, corrected p-value < 0.05 was the uniform threshold for CpGs / genes. However, the corrected p-values are usually not comparable between different analysis strategies. To exclude the possibility that the optimal strategies are cutoff-dependent, the authors should evaluate the robustness of their approaches of selecting optimal strategies upon different cutoffs.

**Reviewer 2**

**Are you able to assess all statistics in the manuscript, including the appropriateness of statistical tests used?**

Yes, and I have assessed the statistics in my report.

**Comments to author:**

The authors compared different strategies and methods for analyzing DNA methylation and RNA-seq data. The paper is well written and provides an interesting and important comparison. However, a few things need to be adressed or clarified.

- In the association models, were the methylation/RNAseq values or the traits used as outcome (i.e. dependent variable)? According to the calculation of HCs (methods part D), it reads as traits were used as exposure for the methylation and as outcome for the RNAseq analyses. For comparing the normalization methods and the statistical models, it would make sense to use the methylation/RNAseq values as dependent variables.

- It seems that the RNAseq analyses were not corrected using Bacon. Can you please explain why, as this is not obvious since Bacon was designed to correct the test statistics in both EWAS and TWAS. Furthermore, please add the inflation and bias estimated by Bacon to Supplementary Table 1.

- Results page 5, line 112: Please change to "D) Technical covariates" to be more specific

- line 129: it should state "supplementary table 1a and figure 2a", and a reference to figure 3a should be added

- line 131: instead of "normalization methods", it should state e.g. "methylation value preprocessing"

- page 6, the results of C) (line 147) are a bit unclear. Does this sentence summarizes the replication for BMI? To what was the Houseman6 similar to, and Houseman3 lower? According to Table S1b, these statements do not relate to the base model.

- line 176: Please add a reference to supplementary table 1b

- line 191: Instead of "not correcting for technical covariates or cell-counts" it should be written "correcting solely for technical covariates or cell-counts, respectively".

- Discussion line 250: It is not obvious how the "HCs still contain confounders which were associated with age" since age was regressed out from the methylation levels already using HC calculation in CATE. How can thus the HCs confound the methylation-age relation, i.e. were the HCs still correlated with age?

- Methods line 325: Were really only probes with >95% missing among the samples excluded, not >5% missing? 95% missing values seem quite high, even though missings were imputed afterwards.

- line 414: The description of the statistical tests applied for RNAseq seems not quite precise. Should it be read "linear model fit of the Limma package (lmFit)" corresponding to Figure 1b (instead Voom)? In addition, there are 5 models included in supplementary table S1b, and what is the difference between lmF and VlmF there?

- Are the PCs calculated in RNAseq part D) correlated with the traits, sex, technical covariates or cell-counts?

- Suppl Table 1b: in column #Meta there appears only "MT", please add the correct numbers. Are the values in columns %repl* really reported as per cent, as they would be very low in this case?

Minor issues:

- Is ref [30] really the correct one for the paragraph on line 84?

- Figure 2: maybe it would increase legibility to re-order the panels by setting the analyses in rows and the traits in columns, and enlarge the figures afterwards

- Figure 3: The Venn diagrams representing the association overlap should be presented for age and BMI, too, similarly to smoking. In addition, re-ordering as suggested for Figure 2 should be considered as the numbers are hard to read in the Venn diagrams.

- Please explain the abbreviations for HC, TC and CC in Figure 2a/b legends where applicable

- Table 1: Please provide also the units of the blood cell-type measurements

**Authors Response**

**Point-by-point responses to the reviewers’ comments:**

Comment

*Response*

Reviewer #1: In this manuscript, the authors evaluated the commonly used analysis strategies for EWAS and TWAS studies, and they tried to give practical guidelines for choosing suitable strategies. I do acknowledge the values of such evaluation studies. However, the value of this study is limited due to the following three major considerations. 

1. The practical guidelines provided in this study are based on the analysis of four cohorts. Those guidelines are valuable only if they can be applied to other datasets. For example, the smoking / non-smoking datasets mentioned in background (ref 16-19). The authors mentioned that using different analytical strategies can partially explain the variation between results from different cohorts. To prove the usefulness of the practical guidelines, the authors could examine whether using the recommended strategies of this study to re-analyze those public datasets can significantly reduce the variation. 

*We thank the reviewer for this suggestion. We feel that our practical guidelines are valid, since we have used 4 separate cohorts and robustly tested the commonly used analysis methods for DNA methylation and RNAseq. Most of the validation studies for DNA methylation and RNAseq analysis methods concentrate on optimizing analyses within 1 cohort, and not on replicability across studies. Therefore, our approach is novel and of great value for population genomics, in which external replication and meta-analysis is essential to identify robust signals. We agree that analyzing additional datasets would be the best way to further prove the impact of a standardized method. However, in order to apply the reviewer’s comment, we would need to have multiple publicly available datasets available, including phenotypes and covariates. We have attempted to collect this information, but to our knowledge, none of the datasets we investigated has individual-level data and phenotypic information publicly available. Most studies deposit summary-level data or DNA methylation/RNAseq profiles without phenotypes. The reviewer’s point highlights that, even though datasets are now more often published online, the lack of corresponding phenotypic information inhibits further (replication) analyses. To our opinion, this further shows the importance of these benchmarking studies and how they can provide some insight into the differences between studies where different methods have already been used, as well as aid in reducing heterogeneity in methods for future studies. We have added a few sentences about this in the discussion section. 

Section: DISCUSSION, Page: 13, Lines: 341-348 
“In this study, we have compared multiple analysis strategies on four cohorts and suggested a base model to reduce heterogeneity between studies. The most ideal validation would be to re-analyze a number of published studies using this optimal model and demonstrate a decrease in heterogeneity between results of previous analyses and those with the new model. However, to our knowledge, for none of the studies we investigated this was possible, due to lack of publically available phenotypic information or lack of publically available individual level DNAm/RNAseq data. As it may not always be possible to share such data publicly, this further shows the need for more standardized DNAm/RNAseq methods, so results between studies can be compared more easily.”* 

2. This study evaluated the optimal analysis strategies for age, BMI and smoking, and obtained distinct strategies. The authors should investigate the causes of such phenotype-dependent optimal strategies. For example, smoking is a binary variable, while BMI is a continuous variable. Whether a strategy is specifically suitable for binary or continuous variables? Why? 

*We agree with the reviewer’s suggestion. In order to investigate whether a strategy is specifically suitable for binary or continuous variables, we further analyzed age and BMI by converting them into categorical variables, i.e. highest vs lowest tertiles. We added the details of these analyses in the methods and results section and briefly discussed these in the discussion. We also added these results to supplemental table 1 and included the plots in supplemental figure 1. 
For DNAm and RNAseq, when we used age/BMI as categorical instead of continuous exposures, the differences between methods remained largely the same. However, the categorical models consistently resulted in lower number and percentage of significantly replicated CpGs/genes as compared to the continuous models, reflecting the lower power of categorical phenotypes. The exception was in the hidden confounders (HCs) correction model for age, where the categorical models resulted in larger number of significantly replicated CpGs/genes as compared to continuous models. Due to the complexity of an exposure like age, it is difficult to interpret this result. It could be that HCs calculated by regressing out continuous age as exposure and other confounders, overcorrect for age, or that HCs calculated by regressing out categorical (0/1) age as exposure do not sufficiently correct for age due to categories and exclusion of individuals from the middle tertile, leading to decrease in power. Overall, these results seem robust for categorical/continuous trait definitions, but do emphasize that HCs correction may be challenging when working with categorical exposures. 
For DNAm, we did not analyze BMI into categorical exposure because the numbers of the significantly replicated CpGs were already small for the continuous models (average of <12 CpGs) when a Bonferroni threshold was used for multiple testing. This made it difficult to draw conclusions when comparing different methods within continuous models, and therefore would have made it even more difficult to compare results between categorical models. We made the following changes to the Methods, Results and Discussion sections: 

Section: METHODS, Page: 19, Lines: 509-518 
“Categorical analyses for age and BMI 
In order to investigate whether an optimal analysis strategy is dependent on whether the independent variable is continuous or categorical, we expanded our association analyses on age and BMI by converting them into tertiles. We used the highest and lowest tertiles to define the categories. The results of these categorical analyses were compared with the results of the continuous analyses where age and BMI were used as continuous measures. For DNAm, we did not analyze BMI into categorical exposure because the numbers of significantly replicated CpGs were already small for the continuous models (average of <12 CpGs) when a Bonferroni threshold was used for multiple testing. This made it difficult to draw conclusions when comparing different methods within continuous models, and therefore would have made it even more difficult to compare results between categorical models.” 

Section: RESULTS, Page: 9, Lines: 238-244 
“Categorical analyses for age and BMI 
For DNAm and RNAseq, when we used age/BMI as categorical instead of continuous exposures, the differences between methods remained largely the same. However, the categorical models consistently resulted in a lower number and percentage of significantly replicated CpGs/genes as compared to the continuous models. The only exception was in the hidden confounders (HCs) correction model for age, where the categorical models resulted in larger number of significantly replicated CpGs/genes as compared to continuous models.” 

Section: DISCUSSION, Page: 12, Lines: 316-328 
“Categorical analyses for age and BMI 
To assess whether strategies are influenced by the continuous or categorical definition of the exposure, we analyzed age and BMI both as continuous and categorical (i.e. highest versus lowest tertiles) exposures of interest. All models responded similarly to the categorical exposure in comparison to the continuous exposure, showing lower number and percentage of replicated signals, indicating lower power for categorical exposures. For both DNAm and RNAseq analyses, we observed differences in performance between models only with HCs correction. The models with 5 HCs for age performed worse when we used age as a categorical variable with highest vs lowest tertiles and excluded the middle tertile. Likely, these results indicate that HCs are insufficiently adjusted for age when it is included as a categorical variable (compared to continuous). Overall, these results seem robust for categorical/continuous exposure definitions, but do emphasize that HCs correction may be challenging when working with categorical exposures.. For continuous variables and most categorical variables (e.g. BMI tertiles and smoking), using HCs performed best and is still recommended.”* 

3. In this manuscript, corrected p-value < 0.05 was the uniform threshold for CpGs / genes. However, the corrected p-values are usually not comparable between different analysis strategies. To exclude the possibility that the optimal strategies are cutoff-dependent, the authors should evaluate the robustness of their approaches of selecting optimal strategies upon different cutoffs. 

*We thank the reviewer for this nice suggestion. As suggested, we compared additional p-value options; FDR threshold (corrected p-value < 0.05), lowest threshold (uncorrected p-value < 0.05) and highest threshold (uncorrected p-value < 1 x 10-8). We added the details of these analyses in the methods and results section and briefly in the discussion. We also added these results to supplemental table 1 and in supplemental figure 2. As expected, less stringent cutoffs led to an increase of replicated signals but at a decreased replicated rate for both DNAm and RNAseq. Most models responded similarly to this change and the respective performance between methods did not change. However, for BMI and smoking in DNAm analysis, the lowest threshold P < 0.05 showed fewer significant CpGs as compared to the other three thresholds. This was caused by the 333-fold increase of significant CpGs in discovery meta-analysis for BMI and 8.6-fold increase for smoking when we used lowest threshold in comparison to FDR threshold. In contrast, the discovery meta-analysis showed only 1.12-fold increase of significant CpGs for age. As a result, the Bonferroni threshold for replication was strongly increased, and most of the previously replicated CpGs did not survive this threshold. For RNA-seq, the previously observed differences between most methods became more pronounced. Overall, the main conclusions did not change. We made the following changes to the manuscript. 

Section: METHODS, Page: 19, Lines: 520-527 
“Evaluation using different p-value cut-offs 
For all the comparisons mentioned, both discovery and replication results were Bonferroni corrected. In addition to using the Bonferroni threshold for the discovery results, we applied three other thresholds to evaluate the robustness of the approaches: (1) Benjamini-Hochberg FDR threshold (corrected p-value <0.05) (2) highest threshold (uncorrected p-value threshold <1x10-8) and (3) lowest threshold (uncorrected p-value threshold <0.05). Differences between models were compared between p-value thresholds to establish that the models show similar (respective) results independent of p-value thresholds.” 

Section: RESULTS, Page: 8, Lines: 219-236 
“Evaluation using different p-value cut-offs 
The results for additional p-value cutoffs (FDR, uncorrected <1x10-8 and uncorrected <0.05) are available in supplemental figure 2 and supplemental table 1. Less stringent cutoffs led to an increase in absolute numbers of replicated signals but at a decreased relative replication rate for both DNAm and RNAseq. Most models responded similarly to this change and the respective performance between methods did not change. 
For BMI and smoking in the DNAm analyses, the lowest threshold P < 0.05 showed fewer significant CpGs as compared to the other three thresholds. This was- caused by a 333-fold increase of significant CpGs in discovery meta-analysis for BMI and an 8.6-fold increase for smoking when we used lowest threshold in comparison to FDR threshold. In contrast, the discovery meta-analysis showed only a 1.12-fold increase of significant CpGs for age. As a result, the Bonferroni threshold for replication was strongly increased, and most of the previously replicated CpGs did not survive this threshold. 
For the normalization options (A) and covariate correction options (D) in RNAseq analyses, the respective differences between the options were unchanged depending on p-value cutoff. For the gene inclusions thresholds (B), it showed that including only the most highly expressed genes yields a slightly higher replication rate using the uncorrected p-value threshold. For the statistical test comparison (C), using lower p-value thresholds (FDR and uncorrected) provided a more pronounced difference between the models.” 

Section: DISCUSSION, Page: 11, Lines: 304-314 
“Evaluation using different p-value cut-offs 
For all models, we observed a balance with more stringent p-value cutoffs resulting in fewer replicated signals, but a larger replication rate. In general, we recommend using Bonferroni corrected p-values with a cutoff of p < 0.05. The FDR corrected p-values can provide an alternative. Decreasing the p-value threshold stringency always leads to increased false positives and thus a lower replication rate. Using uncorrected p-value cutoffs (whether nominal 0.05 or genome-wide 1E-8) is not recommended. 
For DNAm, the differences between methods were similar for all thresholds, and the main conclusions did not change. For RNAseq, these results further show that the GLM and edgeR’s glmQLF models are more conservative (lower number but higher percentage of replicated signals) while limma’s linear model fit is more liberal (higher number but lower percentage of replicated signals) compared to the base model. The LM model is still recommended.* 

Reviewer #2: The authors compared different strategies and methods for analyzing DNA methylation and RNA-seq data. The paper is well written and provides an interesting and important comparison. However, a few things need to be adressed or clarified. 

- In the association models, were the methylation/RNAseq values or the traits used as outcome (i.e. dependent variable)? According to the calculation of HCs (methods part D), it reads as traits were used as exposure for the methylation and as outcome for the RNAseq analyses. For comparing the normalization methods and the statistical models, it would make sense to use the methylation/RNAseq values as dependent variables. 

*The DNA methylation or RNA expression values were used as the outcome (i.e. dependent variable) in all the association analyses. In addition, for the calculation of HCs (methods part D), both DNA methylation and RNAseq were used as outcome and age, BMI or smoking were used as exposures of interest. In the method section D for DNA methylation or RNAseq, the term ‘phenotype’ (or ‘trait’ as referred by the reviewer), refers to the exposures of interest (age, BMI or smoking) and not DNA methylation. We have now changed this term to ‘exposure of interest’ in order to make it clearer. This was done throughout the text.* 

- It seems that the RNAseq analyses were not corrected using Bacon. Can you please explain why, as this is not obvious since Bacon was designed to correct the test statistics in both EWAS and TWAS. Furthermore, please add the inflation and bias estimated by Bacon to Supplementary Table 1. 

*The inflation and bias estimated by Bacon for DNAm are now added to Supplementary Table 1. We did not use the bacon correction for the RNAseq models, as this is not commonly applied to RNA datasets compared to DNA methylation datasets. In our initial selection for the models, we attempted to limit the options to those commonly used in the literature. However, we agree with the reviewer that correcting for test statistics inflation also when analyzing RNAseq data is sensible, and added a comment on this to the discussion. 

Section: DISCUSSION, Page: 11, Lines: 300-302. 
“For RNAseq data, we did not use the Bacon package to correct for inflation of test statistics, as this is not yet widely used for RNAseq datasets. However, applying bacon correction on RNAseq data is becoming more common and should be considered in future RNAseq studies.”* 

- Results page 5, line 112: Please change to "D) Technical covariates" to be more specific 
*This has been changed.* 

- line 129: it should state "supplementary table 1a and figure 2a", and a reference to figure 3a should be added

*This has been changed.* 

- line 131: instead of "normalization methods", it should state e.g. "methylation value preprocessing" 
*This has been changed here and at several other places in the text.* 

- page 6, the results of C) (line 147) are a bit unclear. Does this sentence summarizes the replication for BMI? 
*It summarizes the replication for age. We have now added this more explicitly.* 

- To what was the Houseman6 similar to, and Houseman3 lower? According to Table S1b, these statements do not relate to the base model. 
*We have now added “as compared to the base model” for the sentences related to Houseman6 and Houseman3.* 

- line 176: Please add a reference to supplementary table 1b 
*The reference has been now added.* 

- line 191: Instead of "not correcting for technical covariates or cell-counts" it should be written "correcting solely for technical covariates or cell-counts, respectively". 
*This has been corrected, as per suggestion.* 

- Discussion line 250: It is not obvious how the "HCs still contain confounders which were associated with age" since age was regressed out from the methylation levels already using HC calculation in CATE. How can thus the HCs confound the methylation-age relation, i.e. were the HCs still correlated with age? 
*This is indeed not clear. The issue with age is that it is confounded by, and affects many different other factors. In addition, it is difficult to interpret what exactly is captured in a hidden confounder. Therefore, it is not transparent what the influence of hidden confounder correction is on the age analysis. As the reviewer suggests, the HCs are not correlated with age after the age-adjustment, while our comments in the paper suggested that they might be. We removed this statement from the discussion.* 

- Methods line 325: Were really only probes with >95% missing among the samples excluded, not >5% missing? 95% missing values seem quite high, even though missings were imputed afterwards. 
*This was indeed a typo. We have now corrected “>95%” to “>5%”.* 

- line 414: The description of the statistical tests applied for RNAseq seems not quite precise. Should it be read "linear model fit of the Limma package (lmFit)" corresponding to Figure 1b (instead Voom)? In addition, there are 5 models included in supplementary table S1b, and what is the difference between lmF and VlmF there? 
*In a previous version of our analysis, we experimented with the way to input Voom-transformed data into limma’s linear model fit. What we are calling VlmFit is the one most often used, and we ended up including this one in all comparisons. The “lmFit” method is not referred to anywhere in the paper and is now removed from the supplements to avoid confusion. We used the name “limma” now in all figures and text to avoid confusion.* 

- Are the PCs calculated in RNAseq part D) correlated with the traits, sex, technical covariates or cell-counts? 
*The PCs are indeed correlated with these factors. The correlation seems a bit stronger with the technical covariates and the cell-counts than with age, smoking or BMI. Although PCs could be used when technical factors and cell-counts are not available, we believe that using hidden confounders instead could be more efficient, as it allows for adjusting for the exposures and any known factors. This is also represented in the results, where the HCs perform better than PCs or only technical covariates and/or cell-counts. A few sentences on this were added to the discussion (line 293-300).* 

- Suppl Table 1b: in column #Meta there appears only "MT", please add the correct numbers. Are the values in columns %repl* really reported as per cent, as they would be very low in this case? 
*The column #Meta was added to indicate which meta-analysis method was used; METAL (MT) or GWAMA (GW). As the GWAMA tool requires input that is not provided by some RNA expression statistical methods, we opted to use only METAL for the RNA analysis. (for those where both could be run; the results were identical). This column is redundant and now removed from the table. The values are indeed shown in rate and not percent, this is changed accordingly (also in figure 2b). We added a few sentences on this in the results (lines 214-217).* 

Minor issues: 

- Is ref [30] really the correct one for the paragraph on line 84? 
*It was not the correct one. It is now corrected.* 

- Figure 2: maybe it would increase legibility to re-order the panels by setting the analyses in rows and the traits in columns, and enlarge the figures afterwards. 
*The figures 2a and 2b are now updated as suggested.* 

- Figure 3: The Venn diagrams representing the association overlap should be presented for age and BMI, too, similarly to smoking. In addition, re-ordering as suggested for Figure 2 should be considered as the numbers are hard to read in the Venn diagrams. 
*The venn diagrams are now given for age and BMI, in addition to smoking. In addition, we have re-ordered them as suggested.* 

- Please explain the abbreviations for HC, TC and CC in Figure 2a/b legends where applicable. 
*This has now been explained.* 

- Table 1: Please provide also the units of the blood cell-type measurements. 
*The units are now provided. They are percentages of total cell counts.*

**Second round of review**

**Reviewer 1**

My comments have been addressed.

**Reviewer 2**

I thank the authors for clarifying the issues raised and adding the additional analyses to the manuscript. From my point of view, only a few minor issues remain regarding Supplementary Table 1b, and the newly added paragraphs:

- Results line 173 (RNAseq) and Table S1: Please cross-check the reported mean replication rates for RNAseq with Table S1b as they differ from column P (i.e. 54%, 55%, 51% for age, BMI, smoking). In addition, the values in columns P-T should be multiplied by 100 to represent % as stated in the column headers. The lmFit method is also still included in Table S1.

- line 226: I think it should state "...P < 0.05 showed fewer replicated CpGs..." instead of "significant CpGs"

- line 309: The term "genome-wide" for p<1E-8 is not quite correct and also misleading in this context. I suggest to use e.g. "a too conservative" instead, which reflects a potential over-correction of the results.

- line 523: please change "(corrected p-value<0.05)" to "(FDR<0.05)" as in a strict sense the FDR threshold is not a corrected p-value.

**Authors Response**

**Point-by-point responses to the reviewers’ comments:**

- Results line 173 (RNAseq) and Table S1: Please cross-check the reported mean replication rates for RNAseq with Table S1b as they differ from column P (i.e. 54%, 55%, 51% for age, BMI, smoking). In addition, the values in columns P-T should be multiplied by 100 to represent % as stated in the column headers. The lmFit method is also still included in Table S1.
*These values are checked and the ones from the supplemental table were correct. all other values were checked also to be sure. The lmFit lines have been removed from the table.*

- line 226: I think it should state "...P < 0.05 showed fewer replicated CpGs..." instead of "significant CpGs"

*Agreed and corrected.*

- line 309: The term "genome-wide" for p<1E-8 is not quite correct and also misleading in this context. I suggest to use e.g. "a too conservative" instead, which reflects a potential over-correction of the results.

*Agreed and corrected.*

- line 523: please change "(corrected p-value<0.05)" to "(FDR<0.05)" as in a strict sense the FDR threshold is not a corrected p-value.

*Agreed and corrected.*
